# Can a Like Save the Planet? Comparing Antecedents of and Correlations Between Environmental Liking on Social Media, Money Donation, and Volunteering

**DOI:** 10.3389/fpsyg.2019.01989

**Published:** 2019-08-28

**Authors:** Alexander Georg Büssing, Annelene Thielking, Susanne Menzel

**Affiliations:** Didactics of Biology, Department of Biology/Chemistry, Osnabrück University, Osnabrück, Germany

**Keywords:** social media, pro-environmental behavior, Andean bear, real-world activity, social media for sustainability, wildlife conservation

## Abstract

Due to the societal dissemination of digital technology, people are increasingly experiencing environmental topics through digital media channels such as social networks. Several researchers therefore have proposed these channels as a possibility to strengthen sustainable development based on their cost-efficient nature. But while prior studies have investigated isolated factors for understanding environmental social media behavior, there is still scarce understanding of the relevant underlying motivational factors and possible connections with more traditional environmental behaviors. Therefore, the present study applied the established socio-psychological model of goal-directed behavior and compared the desires for liking as a fundamental form of digital social media behavior with the desires for two traditional environmental behaviors (money donation and volunteering) in a cross-sectional research design. Within the biodiversity conservation case of the Andean bear (*Tremarctos ornatus*) in Ecuador, we compared the antecedents for the desires for the corresponding environmental behaviors (RQ_1_) as well as their connections between each other (RQ_2_). Within a sample of 407 Ecuadorian students (*M*_age_ = 20.94 years, *SD* = 2.25, 61.2% female), we found the marginal effect of perceived behavioral control on the desires for liking on social media as the main difference concerning the antecedents of the behaviors because money donation and volunteering largely depended on personal resources such as time or money. Furthermore, gender emerged as the second main difference between the behaviors due to it only being predictive for the liking motivation. Enjoyment was the only variable that coherently predicted all three behaviors. Finally, desires for liking on social media predicted both other behaviors in robust regression analyses, but were only predictive for volunteering in corresponding path models. The results illustrate how cost-efficient digital environmental behaviors such as liking may be suitable for sparking low-level environmental action, which may entail more pronounced forms of environmental activism, at least when they involve feasible personal costs. Overall, the findings are in line with prior research regarding the less demanding nature of liking, but further elaborate on the importance of gender for digital environmental behavior and correlates between digital and classical environmental behaviors.

## Introduction

While direct nature experiences are decreasing (Soga and Gaston, 2016), people experience social media sites as a source of information about environmental topics (Cheung et al., 2015). Based on this development, researchers have begun to investigate the role of new media channels for purposes of nature conservation. Inspired by studies from the political sciences that have demonstrated how social media usage may contribute to political activism (Boulianne, 2015; Masías et al., 2018), several researchers proposed social media as a tool for education about and participation in environmental issues (Pearson et al., 2016). To investigate the possibilities of environmental social media usage, several studies have utilized existing social networking data to investigate content, places, or agents of environmental posts (Di Minin et al., 2015). But based on their *post hoc* nature and dependence on publicly available data (van der Sloot et al., 2016), these analyses often stay isolated on specific factors. This isolation may neglect the variety of motivationally relevant psychological factors for environmental behavior (Gifford and Nilsson, 2014), explaining why environmental psychologists still have scarce understanding about environmental behavior in virtual environments (Gifford, 2014).

Such an understanding is required to strengthen sustainable development because environmental degradation is relentlessly proceeding, particularly concerning the integrity of biodiversity (Steffen et al., 2015). This degradation is particularly problematic in biodiversity hotspots such as the tropical Andes (Myers et al., 2000). In this and other regions, large mammals including the Andean bear (*Tremarctos ornatus*) are at risk to become extinct for example due to steady deforestation for human economic development or illegal hunting in reaction to livestock killings (Garcia-Rangel, 2012). Due to these developments, the species was predicted to be the third most threatened carnivore species to become extinct and should therefore be prioritized in conservation efforts (Cardillo et al., 2004). To increase the awareness on the respective issue, environmental organizations such as the *Andean Bear Foundation* (Fundación Oso Andino) are attempting to promote conservation-related activities on Facebook^1^ and Twitter^2^. As several studies have demonstrated, environmental organizations rarely utilize the full potential of these new communication channels and neglect for example the possibility to dialogically interact with stakeholders or foster engagement with their objectives (Lee et al., 2018).

But to increase peoples’ involvement with environmental content on social media, environmental organizations and practitioners require further knowledge about determining factors of environmental social media behavior (Aksoy et al., 2013). Contrary to other environmental behaviors such as volunteering (Bruyere and Rappe, 2007) or money donation (Ku and Zaroff, 2014), there is only scarce theoretical and empirical knowledge about the psychological determinants of social media behavior in general and environmental social media behavior in particular (Büscher, 2016; Cihon and Yasseri, 2016). Therefore, this paper aims at a better understanding of environmental behavior in social media from a psychological perspective. For this purpose, we concentrated on two main research foci.

First, we selected the relevant behavior of environmental liking on social media and compared the motivational antecedents for this environmental social media behavior with two more classical behaviors within the selected biodiversity conservation context of the Andean bear. Results concerning this aim may explicate theoretical similarities and differences between the behaviors, an explanation which later could aid conservation practice in an application perspective. Based on their good theoretical understanding, we selected money donation and volunteering as classical behaviors and propose the following first research question:

RQ_1_: Which motivational factors affect environmental liking on social media compared to the classical environmental behaviors of money donation and volunteering?

Besides a comparison of antecedents for the selected behaviors, prior studies were unable to investigate the connections between environmental behavior in social media and classical real-world behaviors because these studies most often concentrated on one of both types of environmental behaviors. To better understand how these behaviors are connected with each other, we investigated the possibility of environmental liking as a pathway behavior for more effortful behaviors such as money donation and volunteering as a second research aim. Several studies have illustrated how social media requires only few personal resources (Baker and White, 2010), which could facilitate participation within environmental topics (Pearson et al., 2016). But while these studies were of a rather general nature, these findings have not yet been transferred to environmental behavior. Within this aim, we addressed the following second research question:

RQ_2_: What are the connections between environmental liking on social media and the classical environmental behaviors of money donation as well as volunteering?

To further underpin our investigation and hypothesize possible motivational antecedents for the behaviors (RQ_1_) as well as correlations between them (RQ_2_), we utilized the general socio-psychological *model of goal-directed behavior* (MGB) following Perugini and Bagozzi (2001) and adapted this model to the behaviors under study.

### Theoretical Framework and Hypotheses

#### Antecedents of Environmental Behavior

Generally, *social media* represents a collective term for a variety of web-based services, which allow their users to create public or non-public profiles to connect with other people or organizations (Boyd and Ellison, 2007). Whereas individuals may engage in social media through different activities such as liking, sharing or posting content (Aksoy et al., 2013), likes can be described as the building blocks of social media because every digital network relies on their users’ ability to indicate affective responses to specific content (van Dijck and Poell, 2013). While a variety of different indicators may be utilized to communicate these affective responses, liking generally describes the process of pressing a like button below particular posts.

Therefore, liking emerges as the foundation of digital societal action due to the fact that all further spread of information needs to trigger positive reactions in specific networks to cause the multiplication of content (Lipsman et al., 2012). This triggering is particularly relevant for environmental topics because environmental activists might use social media for spreading information to draw attention or increase vital knowledge (Pearson et al., 2016).

Despite the importance within environmental issues and their diffusion in everyday life, there is only relatively scarce theoretical understanding of new media behaviors such as liking in environmental topics (Büscher, 2016). Because environmental psychologists have a long history in applying different theoretical models (Osbaldiston, 2013), the adaptation of classical psychological theory may assist the further theorization of new media environmental behavior and allow for a comparison to more classical environmental behaviors.

One of the theories that is suitable for assisting a further theory of environmental social media behavior is the MGB (Perugini and Bagozzi, 2001). This model builds on the *theory of planned behavior* (TPB; Ajzen, 1991), and adds emotions as well as past behavior as additional predictors. Furthermore, the model proposes desires as a mediator of the independent variables on the behavioral intentions, which represented the initial dependent variable in the TPB-framework (Perugini and Bagozzi, 2004).

*Desires* describe the “motivational state of mind wherein appraisals and reasons to act are transformed into a motivation to do so” (Perugini and Bagozzi, 2001). While intentions are more concretely connected to actions at specific time points (Perugini and Bagozzi, 2004), desires aim for the general motivation to fulfill the selected behaviors. Based on our research aims, which concentrated on the antecedents for the general motivation (RQ_1_) as well as the correlations between them (RQ_2_), we concentrated on desires as the dependent variable.

Concerning the independent variables, the MGB includes attitudes, subjective norms, and perceived behavioral control as independent variables consistent with the TPB. *Attitudes* represent either a positive or negative psychological evaluation of an object or a context (Ajzen, 1991). In the present study, such an evaluation concentrates on the protection of the Andean bear and indicates the compliance to this aim. *Subjective norms* can be understood as the perceived social pressure to fulfill a specific behavior and refer to individual normative beliefs (Ajzen, 1991). For the intended behavior, this definition would describe the perceived social pressure of protecting the Andean bear. These norms are stable across online and offline contexts (Postmes et al., 2000) and were connected to social media behavior in prior studies (Pelling and White, 2009; Baker and White, 2010; Hilverda et al., 2018). Furthermore, a randomized experiment found social influences as a major contributor to online and real-world political mobilization (Bond et al., 2012). Finally, *perceived behavioral control* as the last predictor from the TPB refers to the perceived ability to perform a specific behavior (Ajzen, 2002). In the present study, this predictor entails the personal internal and external resources to protect the Andean bear. As described above, this factor was demonstrated to be not a major predictor in contexts of social media (Pelling and White, 2009; Baker and White, 2010), but may be of high importance for the other environmental behaviors of donation and volunteering (Gifford and Nilsson, 2014). Based on these independent variables, we propose our first three hypotheses:

H_1_: Attitudes will be a predictor variable for the desires for environmental liking on social media as well as donating money and volunteering.

H_2_: Subjective norms will be a predictor variable for the desires for environmental liking on social media as well as donating money and volunteering.

H_3_: Perceived behavioral control will only be a predictor variable for the desires for donating money and volunteering, but not for environmental liking on social media.

Besides these variables originating from the TPB, the MGB integrates anticipated emotions as well as past behavior into the framework. As prior studies have shown, these variables affect social media as well as environmental motivations (Pelling and White, 2009; Song et al., 2012; Passafaro et al., 2014), which is why the selection of the MGB seems interesting for the research questions at study.

Emotions can generally be described as multidimensional and short lasting affective reactions to specific stimuli (Scherer, 2005). In the present study, this stimulus would be the protection of the Andean bear. Whereas emotions can be viewed as a dimensional phenomenon based on the dimensions of value (positive/negative) and arousal (high/low), prior studies in wildlife psychology have demonstrated improved measurement results and a better interpretability when emotions are viewed from a discrete emotion perspective (Scherer, 2005; Jacobs et al., 2012). In this view, specific discrete emotions can be differentiated by their cognitive causes and affective experience (Izard, 2007).

In the present study, we concentrated on such a discrete approach and selected enjoyment and anger as independent variables because these emotions have been shown to be connected to wildlife protection behavior in prior studies (Vaske et al., 2013; Jacobs et al., 2014). *Enjoyment* describes the positive emotional perception in positively valued situations with a medium amount of control (Scherer, 2005). For example, people will experience enjoyment when they evaluate concrete situations such as building fences or collecting donations as being positive. While also involving a medium amount of control, *anger* is elicited in negatively valued events and is also based on frustration (Scherer, 2005; Kuppens et al., 2008). In the present context, anger would be elicited if the participant is generally opposed to protect the species. As a final variable from the MGB, *past behavior* reflects the past fulfillment of the specific behavior (Perugini and Bagozzi, 2001). We also included this variable in our model because this variable has shown predictive effects on other environmental behaviors including recycling or mobility decisions (Carrus et al., 2008).

H_4_: Enjoyment will be a positive predictor variable for the desires for environmental liking on social media as well as donating money and volunteering.

H_5_: Anger will be a negative predictor variable for the desires for environmental liking on social media as well as donating money and volunteering.

H_6_: Past behavior will be a predictor variable for the desires for environmental liking on social media as well as donating money and volunteering.

Besides these independent variables from the MGB, we added age and gender as demographic control variables to our models due to the fact that prior studies indicated effects on pro-environmental (Olsson and Gericke, 2017) as well as social media behavior (Schwartz et al., 2013; Musil et al., 2017). In these studies, female and older people showed higher environmental orientations (Gifford and Nilsson, 2014). Concerning their connections with the dependent variables, we propose our final two hypotheses for our first research question:

H_7_: Female participants will show higher desires for environmental liking on social media as well as donating money and volunteering.

H_8_: Older people will show higher desires for environmental liking on social media as well as donating money and volunteering.

**FIGURE 1:**
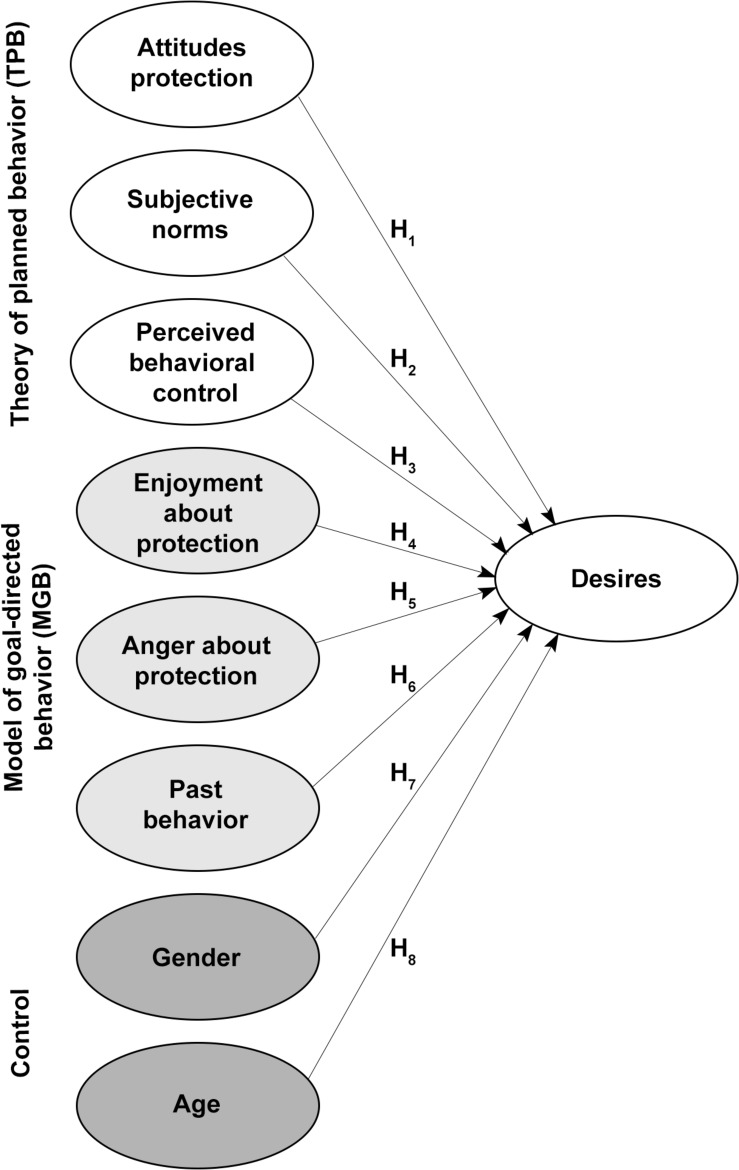
Overview of the independent variables originating from the theory of planned behavior (TPB; white variables), model of goal-directed behavior (MGB; light gray variables) and further demographic control variables (dark gray variables), as well as desires as dependent variable.

An overview of all independent variables of our full theoretical model is displayed in Figure 1.

#### Correlations Between Environmental Behaviors

As described above, social media may be used as a way to engage individuals in subsequent activist behaviors, an approach which may be of importance to foster society’s sustainable development (Warren et al., 2014; Pearson et al., 2016). Activists and organizations may engage people due to the informational, relational, or experiential function of social media for environmental behavior (Ballew et al., 2015). Hence, people could be motivated by specific information, social ties to other people, or specific contextually relevant experiences within social media sites (Ballew et al., 2015). But in prior studies, environmental behavior in social media has only been marginally investigated.

Because we selected the three behaviors of liking, money donation, and volunteering, we first investigated possible correlations between the desires for these three behaviors. Based on prior studies, behaviors from similar contexts aiming at the area of conservation should be positively correlated with each other (Kaiser and Wilson, 2004), which is why we propose the following first hypothesis for our second research question:

H_9_: The desires for environmental liking on social media, money donation and volunteering are positively correlated with each other.

Given the hypothesized differences between the behaviors (H_1–8_), we nonetheless also propose differences in these correlations. Money donation as well as volunteering may be described as “classical” behaviors, which also both require a larger amount of personal resources such as money or free time (Leliveld and Risselada, 2017). Therefore, these behaviors should be more strongly correlated with each other than to environmental liking, reflecting these similarities and differences between the behaviors.

H_10_: The desires for money donation and volunteering are more strongly correlated with each other than to the desires for environmental liking on social media.

As described above, we hypothesized a requirement for fewer personal resources than other environmental behaviors for the desires for liking on social media (H_3_). Therefore, liking may generally be easy and efficient for the specific purpose of assisting conservation practice (Baker and White, 2010; Pearson et al., 2016). In particular, because other environmental behaviors heavily rely on personal resources such as money for donations (Leliveld and Risselada, 2017) or time for volunteering activities (Bruyere and Rappe, 2007), social media may be an easy way of “doing something good.”

As prior studies from the context of political mobilization have demonstrated, social media may nonetheless lead to further, subsequent behaviors (Gil de Zúñiga et al., 2012). Within the context of environmental psychology, the triggering of more difficult environmental behaviors has been called *spillover effect* (Nilsson et al., 2017). Given the hypothesized easy and efficient nature of environmental liking, performing this behavior may entail an increased desire for performing other, more difficult environmental behaviors including money donation or volunteering (Ballew et al., 2015).

Such spillover effects depend on several factors, such as similarity between the behaviors or the resources required for the specific behaviors (Margetts and Kashima, 2017). Whereas money donation and volunteering both may be highly similar to each other, money donation may nonetheless be the behavior with the highest difficulty, due to the need for money as a scarce personal resource (Leliveld and Risselada, 2017). Therefore, we hypothesize a possible behavioral spillover effect for environmental liking on social media for volunteering, but not for money donation:

H_11_: The desires for environmental liking on social media will be a positive predictor of the desires for volunteering, but not for the desires for donating money.

## Materials and Methods

### Research Design

The description of environmental linking on social media and the investigation of possible determinants may benefit from using approved methodologies from other social science fields such as environmental psychology (Abbasi et al., 2012). Possible methodologies include survey data, which already were applied to describe general social media usage (Hughes et al., 2012). Such approaches could complement existing knowledge regarding digital conservation behavior and lay the foundation for further experimental investigations.

To compare the differences and correlations between specific variables, we therefore selected a cross-sectional quantitative research design, based on a paper-and-pencil questionnaire. This study design allows a comparison of the different behaviors in an efficient manner. Prior studies have shown the usability of such a design to test the stated research hypotheses and questions (Yun and Trumbo, 2000).

### Sample

Because we selected the context of the Andean bear in Ecuador, we gathered data in June and July 2016 at two universities in Cuenca, Ecuador. We chose the study sites based on the occurrence of the Andean bear because this region is located in proximity to the habitat of the species (Goldstein et al., 2008). Overall, 407 students participated in the survey [249 females (61.2%), *M*_age_ = 20.96 years, *SD*_age_ = 2.24 years, range = 18–34 years]. We decided to collect data from young people because older people use social media sites far less than young people. Therefore, this group may reflect the most important user group of social media (Aksoy et al., 2013). Whereas future studies should expand the range or explicitly investigate the relevance of age for environmental social media usage, the participating students came from diverse disciplines of study, including the life sciences, architecture, economics, psychology, or tourism. The sample nonetheless represents a convenience sample because we did not apply any further randomization.

The study was carried out in accordance with the relevant national guidelines and laws of the study country, the selected university, the Declaration of Helsinki as well as APA’s Code of Conduct to ensure compliance to all ethical and legal standards. This assurance included for example the guarantee of anonymity and participation on a voluntary basis because all participants had the opportunity to decline their participation at any time without negative consequences. Furthermore, we assured informed consent by giving written information on the first page and verbal information prior to the sampling. We therefore ensured consensus about the purpose and aims of the study between all participants and implied this informed consent by survey completion (Nijhawan et al., 2013). Due to the non-medical background, the absence of personal risks, and the full awareness regarding the purpose and aims of all participants, ethics approval was not required by institutional, national, and international guidelines (Nijhawan et al., 2013).

### Questionnaire

#### Overall Design

The questionnaire began with questions regarding demographic data, followed by the psychological scales from the MGB. As demographic data, we asked for the age, gender, as well as the intended degree. Whereas age and intended degree were open questions, gender was asked in a closed format and coded with 1 (female) and 2 (male). We also assessed deeper personality factors related to wildlife protection, which were not analyzed in the present paper. The available data set for the replication of our analyses also excludes these further variables as well as the intended degree in order to protect the anonymity of the participants.

The questionnaire was distributed in Spanish and was constructed by double translation from approved English scales (Song et al., 2012). After the first translation, a different person translated the questionnaire back from Spanish to English, to check the accuracy of the translation. As a final step, the revised Spanish version was discussed with a native Ecuadorian speaker and tested with several students to ensure comprehension. In this paper, we report the English translations of this revised and finally applied version.

All variables except for past behavior were tested by multiple items to enhance the validity of the constructs (Bryman, 2008). If not described differently, all items were measured on a six-point Likert scale, ranging from 1 (*do not agree at all*) to 6 (*agree completely*) and were worded as statements to allow the construction of a Likert scale (Bryman, 2008). The English as well as Spanish wording of all items can be viewed in the Supplementary Material.

#### Model of Goal-Directed Behavior (MGB)

As described in the theoretical background, the MGB proposes attitudes, emotions, subjective norms, perceived behavioral control, as well as past behavior as predictors of desires (Perugini and Bagozzi, 2001). We constructed the items for these distinct scales by using existing MGB scales (Song et al., 2012) and adapting them to the protection of the Andean bear. We aimed to keep the adapted version close to the original version by only replacing the respective objects of the sentences with “the protection of the Andean bear.”

Based on the definition of attitudes, we asked participants to rate their agreement with the belief that the protection of the Andean bear is good. We constructed three items based on the existing scale of Song et al. (2012). We decided to measure discrete emotions with the established Differential Emotions Scale (Izard et al., 1993). The emotions of enjoyment and anger were measured with three items each, and participants were asked to rate their agreement with the items based on the introductory text “If I promoted the protection of the Andean bear, I would feel…” Concerning the variable of subjective norms, we asked participants to rate their agreement if the majority of people would view the protection of the Andean bear as being favorable behavior. The variable of perceived behavioral control asked for the personal belief about being able to support the protection of the species.

The factor of past behavior was assessed using one item for every tested behavior. The items directly asked about the previous performance of the respective behavior. After the introductory text “How often did you…,” participants were asked to rate their agreement to questions concerning all three behaviors. In difference to the other constructs, the scale for past behavior ranged on a five-point scale from 1 (never), 2 (rarely), 3 (occasionally), 4 (often), to 5 (very often).

Finally, desires as the dependent variables represent the motivation to perform the respective behavior (Perugini and Bagozzi, 2001). Based on their lower action connectedness, desires are conceptually different to intentions (Perugini and Bagozzi, 2004). We were mainly interested in measuring the antecedents of the selected protection motivations, and we therefore decided not to further differentiate between desires and intentions. We also decided for the general term of “social network” to ensure comprehension by every participant because some of the participants may not have been part of a specific network, an issue which might have conflicted measurement. We therefore did not differentiate between specific platforms because such differentiation was not part of our intended research questions (Waterloo et al., 2018). All items for the desires were randomized to gain better measurement results and maintain the attention of the participants at a high level.

Besides not testing specific platforms, we also did not measure the independent variables as concretely referring to the behaviors under study as it was proposed by Ajzen (1991), who insisted on measuring the independent variables on the same level of abstraction as the corresponding behavior (“correspondence principle”). Following this principle, all variables should aim for the specific behavior, an approach which would have entailed measuring all independent variables for the behaviors of environmental liking on social media, money donation, as well as volunteering. In prior studies, such a specific strategy proved to entail the best results concerning the measurement of specific behaviors (Kaiser and Wilson, 2004). Violating the correspondence principle may lead to smaller connections, due to the higher specificity between the behaviors and corresponding predictors (Ajzen, 1991).

For the present study, we took this risk because prior research has demonstrated how the correspondence principle may also entail negative consequences, such as the inflation of predictive abilities by variables, including perceived behavioral control (Kaiser et al., 2007). Therefore, a more specific way of measuring for example perceived behavioral control in our study could have confounded the possibility of comparing the effects of the independent variables on the specific behaviors. Furthermore, the violation of the correspondence principle concerned all dependent variables equally. All presented results nonetheless represent rather conservative estimations of possible correlates due to the fact that items following the correspondence principle would yield even stronger connections.

### Statistical Analyses

#### General Procedure

As a first step, we performed a confirmatory factor analysis (CFA) with the latent variables specified by three indicators per factor to ensure the discriminant validity of the scales (Brown, 2015). For this step, we calculated an eight-factor-model with all indicators from the measured variables. Besides evaluating alternative models as another external validity criterion, we also inspected Cronbach’s Alpha as a measure of internal consistency of the variables.

After this evaluation of the measurement model, we tested the predictive abilities of the independent variables from the MGB (H_1–8_) with robust regression analyses. For these regression analyses, we used different sets of independent variables to test which of the independent variables may predict the desires for the three dependent variables of liking, money donation, and volunteering.

We then proceeded by comparing the correlations between the desires (H_9_ and H_10_). To investigate the possibility of a behavioral spillover from environmental liking on money donation and volunteering (H_11_), we calculated two path models, investigating whether the desires for environmental liking on social media may be a predictor for the desires of the both more difficult environmental behaviors (model 1), or there may be a spillover effect on the desires for volunteering, but not for the desires on donating money (model 2). The independent variables were selected based on the results of the preceding regression analysis, excluding variables without predictive abilities for the dependent variables.

We decided to use robust statistical methods because most of the scales were skewed and not normally distributed (see also Table 1). These methods include the estimation of the CFA and the path model with a robust Maximum-likelihood estimator and the selection of Spearman-rho as a correlation coefficient. We did not exclude any cases or impute any data because the selected procedures are robust against such violations of general assumptions but preserve the initially collected data (Field and Wilcox, 2017). Besides the utilization of robust estimators, we used robust versions of ANOVAs, *t*-tests, and regressions. All calculations were done in R-Studio Version 1.1.456 running R Version 3.5.1 (R Core Team, 2018). The script with the applied statistical calculations is available in the Supplementary Material for the replication of the analysis.

**TABLE 1:** Overview of the correlations (Spearman-rho) between the variables with bootstrapped upper and lower 95% confidence intervals above the diagonal and descriptive statistics.

**Variable**	**1**	**2**	**3**	**4**	**5**	**6**	**7**	**8**	**9**	**10**	**11**	**12**	**13**
(1) Age	–	−0.03,0.18	−0.11,0.06	−0.13,0.08	−0.05,0.14	−0.05,0.13	−0.16,0.03	−0.15,0.04	−0.03,0.14	0.04,0.20	−0.08,0.11	−0.20,−0.01	−0.21,0.00
(2) Gender	0.08	–	−0.23,−0.01	−0.21,−0.01	−0.01,0.19	−0.15,0.06	−0.16,0.04	−0.13,0.06	−0.09,0.09	−0.04,0.15	−0.28,−0.09	−0.11,0.08	−0.19,0.01
(3) Attitudes	−0.03	−0.12^∗^	–	0.29,0.46	−0.43,−0.24	0.39,0.54	0.26,0.44	−0.07,0.14	−0.07,0.13	−0.12,0.10	0.22,0.41	0.13,0.31	0.22,0.40
(4) Enjoyment	−0.02	−0.12^∗^	0.38^∗∗^	–	−0.40,−0.24	0.34,0.52	0.28,0.45	−0.12,0.08	−0.07,0.13	−0.10,0.12	0.25,0.45	0.24,0.43	0.33,0.50
(5) Anger	0.05	0.09	−0.34^∗∗^	−0.32^∗∗^	–	−0.40,−0.23	−0.33,−0.15	−0.09,0.10	−0.12,0.11	−0.16,0.03	−0.41,−0.22	−0.26,−0.09	−0.29,−0.09
(6) Subjective norms	0.05	−0.05	0.47^∗∗^	0.44^∗∗^	−0.32^∗∗^	–	0.32,0.50	−0.07,0.15	−0.06,0.13	−0.05,0.15	0.24,0.44	0.17,0.37	0.24,0.44
(7) PBC	−0.06	−0.06	0.35^∗∗^	0.37^∗∗^	−0.24^∗∗^	0.41^∗∗^	–	0.11,0.29	0.02,0.22	0.00,0.20	0.17,0.35	0.43,0.59	0.48,0.63
(8) Past liking	−0.04	−0.04	0.04	−0.02	0.00	0.03	0.20^∗∗^	−	0.21,0.41	0.03,0.26	0.02,0.20	0.04,0.24	0.14,0.32
(9) Past donation	0.06	0.00	0.03	0.04	−0.01	0.04	0.13^∗^	0.31^∗∗^	–	0.30,0.68	−0.08,0.11	0.07,0.22	0.04,0.21
(10) Past volunteering	0.12^∗^	0.06	−0.02	0.01	−0.06	0.04	0.10^∗^	0.14^∗∗^	0.50^∗∗^	–	−0.04,0.15	0.05,0.23	0.02,0.17
(11) Liking	0.02	−0.20^∗∗^	0.32^∗∗^	0.36^∗∗^	−0.31^∗∗^	0.34^∗∗^	0.26^∗∗^	0.11^∗^	0.01	0.05	–	0.18,0.35	0.25,0.43
(12) Donation	−0.09	−0.02	0.22^∗∗^	0.34^∗∗^	−0.18^∗∗^	0.27^∗∗^	0.52^∗∗^	0.14^∗^	0.15^∗∗^	0.14^∗∗^	0.26^∗∗^	–	0.48,0.63
(13) Volunteering	−0.10	−0.09	0.32^∗∗^	0.42^∗∗^	−0.19^∗∗^	0.35^∗∗^	0.57^∗∗^	0.22^∗∗^	0.13^∗^	0.09	0.35^∗∗^	0.56^∗∗^	–
Items	1	1	3	3	3	3	3	1	1	1	3	3	3
Mean	20.96	–	5.64	4.99	1.41	5.12	4.21	1.80	1.11	1.11	5.10	3.97	4.40
*SD*	2.24	–	0.65	0.89	0.67	0.87	0.94	1.18	0.50	0.50	1.13	1.17	1.13
Median	21.00	–	6.00	5.00	1.00	5.00	4.33	1.00	1.00	1.00	5.33	4.00	4.33
α	–	–	0.91	0.69	0.92	0.90	0.72	–	–	–	0.95	0.93	0.91

*Gender was coded as female (1) and male (2). ^∗^*p* < 0.05, ^∗∗^*p* < 0.01. PBC, perceived behavioral control; SD, standard deviation; α, Cronbach’s alpha.*

#### Measurement Results

Kline (2016) recommends the evaluation of model fit by combining the fit indices of the root mean square error of approximation (RMSEA), Bentler comparative fit index (CFI), and the standardized root mean square residual (SRMR). Therefore, we assessed a good model fit in our study by a RMSEA under or equal to 0.05, a CFI over or equal to 0.95, and a SRMR under or equal to 0.05 (Li-tze and Bentler, 1999).

Based on these criteria, the estimated CFA of the theoretical model led to the best and overall good model fit (RMSEA = 0.04, CFI = 0.98, SRMR = 0.04). We also estimated similar models based on correlations between the variables to inspect whether another factor solution might be a better fit to the data.

As displayed in Table 2, no model showed better fit than the initial and theoretically justified model. The alternative models were selected to rule out specific measurement problems like the missing discriminability between money donation and volunteering (alternative model 1) or the existence of one general motivation behind all measured desires (alternative model 3). Because all variables also demonstrated good measurement reliabilities based on Cronbach’s Alpha (Table 1), we continued with further analyses.

**TABLE 2:** Overview of measurement results from the confirmatory factor analysis (CFA) of the fit between the theoretical and two alternative models based on the root mean square error of approximation (RMSEA), Bentler comparative fit index (CFI) as well as standardized root mean square residual (SRMR).

	**RMSEA**	**CFI**	**SRMR**
Theoretical model	0.04	0.98	0.04
Alternative model 1: Classical desires together (^DON^ + ^VOL^)	0.07	0.93	0.05
Alternative model 2: Classical desires (^DON^ + ^VOL^) + Perceived behavioral control together	0.09	0.85	0.07
Alternative model 3: All desires together (^LIKE^ + ^DON^ + ^VOL^)	0.13	0.73	0.10

*^*LIKE*^ = Desire to like for the protection of the Andean bear, ^*DON*^ = Desire to donate money for the protection of the Andean bear, ^*VOL*^ = Desire to volunteer for the protection of the Andean bear.*

## Results

### Correlations and Descriptive Statistics

We found several bivariate correlations between the independent and dependent variables. As described in Table 1, all independent variables from the MGB were significantly correlated with all three desires with a small or medium effect size. While attitudes, enjoyment, and subjective norms showed similar correlations to all three dependent desires, anger was more strongly correlated with the desires for liking (*r* = −0.31, *p* < 0.01) than with the desires for money donation (*r* = −0.18, *p* < 0.01) or volunteering (*r* = −0.19, *p* < 0.01). Differences in the correlations also emerged for perceived behavioral control, which was correlated with a small effect size to the desires for environmental liking (*r* = 0.26, *p* < 0.01), but with a large effect size with the desires for money donation (*r* = 0.52, *p* < 0.01) and volunteering (*r* = 0.57, *p* < 0.01).

Besides these differing correlations of the independent and dependent variables between the desires, we also investigated possible mean differences between the reported desires using robust versions of between groups ANOVAs and *post hoc* robust *t*-tests. Overall, we found the highest motivation for the liking on social media (*M* = 5.10, *SD* = 1.13, *Mdn* = 5.33), followed by the motivation to volunteer (*M* = 4.40, *SD* = 1.13, *Mdn* = 4.33), and the motivation to donate money (*M* = 3.97, *SD* = 1.17, *Mdn* = 4.00). These differences were also statistically significant, as the between-groups ANOVA as well as the *post hoc* comparisons showed. While the motivation to donate money and to volunteer only differed with a small effect size [t*_*DON–VOL*_*(237) = −6.07, *d* = 0.28, *p* < 0.001], we found large effect sizes for the differences between the motivation to like and to donate [t*_*LIKE–DON*_*(235) = 17.30, *d* = 1.04, *p* < 0.001] as well as to volunteer [t*_*LIKE–VOL*_*(237) = 14.70, *d* = 0.83, *p* < 0.001].

The results for the past behaviors resembled this pattern, with the participants reporting the highest past behavior for liking (*M* = 1.80, *SD* = 1.18, *Mdn* = 1.00). Past donation and volunteering behavior were only marginally reported with similar distributions (*M* = 1.11, *SD* = 0.50, *Mdn* = 1.00). These differences were underlined by the statistical tests, which only found differences between Table 1, which only found differences between the past liking behavior and the classical environmental behaviors with small effect sizes [t*_*PASTLIKE–PASTDON*_*(240) = 5.53, *d* = 0.31, *p* < 0.001; t*_*PASTLIKE–PASTVOL*_*(240) = 5.53, *d* = 0.31, *p* < 0.001], but not between the two classical environmental behaviors [t*_*PASTDON–PASTVOL*_*(240) = 0.00, *d* = 0.00, *p* > 0.05].

### Motivational Antecedents (H_1–8_)

Although we found several correlations between the variables (see also Table 1), we mainly interpreted the robust regressions for the correlations between the independent variables and the motivation for liking on social media. To strengthen the theoretical perspective, we utilized a step-wise approach to these regressions. Hence, we first investigated the traditional predictor variables of the TPB (Model 1), followed by the predictor set of the MGB (model 2). In model 3, we selected only the desires for environmental liking as a predictor of money donation and volunteering. Finally, model 4 includes all possible predictors and the full model. Table 3 presents the results of these regressions.

**TABLE 3:** Standardized regression results (β) from the robust regressions for the prediction of the motivations to protect the Andean bear with liking on social media, money donation, and volunteering based on the independent variables of the theory of planned behavior (Model 1: TPB), model of goal-directed behavior (Model 2: MGB), the motivation to like on social media (Model 3: Liking) and the full model with all predictors (Model 4: Full model).

	**Liking**	**Donation**	**Volunteering**
**Model 1: TPB**			
Attitudes	0.48^∗∗∗^	0.03	0.11
Subjective norms	0.16^∗^	0.07	0.12
PBC	0.14^∗^	0.68^∗∗∗^	0.69^∗∗∗^
Adjusted *R*^2^	0.27	0.37	0.43
**Model 2: MGB**			
Attitudes	0.35^∗^	–0.03	0.03
Subjective norms	0.07	0.04	0.05
PBC	0.11	0.59^∗∗∗^	0.61^∗∗∗^
Enjoyment	0.22^∗∗^	0.24^∗∗∗^	0.30^∗∗∗^
Anger	–0.13	0.03	0.04
Past behavior	0.05	0.20^∗∗∗^	0.12^∗^
Adjusted *R*^2^	0.31	0.40	0.45
**Model 3: Liking**			
Liking	−	0.11^∗∗^	0.19^∗∗^
Donation	0.07	−	0.61^∗∗∗^
Volunteering	0.25^∗∗∗^	0.68^∗∗∗^	−
Adjusted *R*^2^	0.14	0.54	0.51
**Model 4: Full model**			
Attitudes	0.34^∗∗^	–0.06	–0.04
Subject. norms	0.08	0.03	0.03
PBC	0.14^∗^	0.55^∗∗∗^	0.58^∗∗∗^
Enjoyment	0.21^∗∗^	0.19^∗∗∗^	0.25^∗∗∗^
Anger	–0.11	0.02	0.04
Past behavior	0.05	0.20^∗∗∗^	^OUT^
Liking	−	0.14^∗∗^	0.17^∗∗∗^
Age	0.02	–0.04	–0.04
Gender	–0.32^∗∗∗^	0.09	–0.10
Adjusted *R*^2^	0.35	0.42	0.48

*Gender was coded with female (1) and male (2). ^∗^*p* < 0.05, ^∗∗^*p* < 0.01, ^∗∗∗^*p* < 0.001. ^*OUT*^ = Variable excluded due to outliers. *R*^2^ = Explained variance.*

Concerning the first regression step (model 1), we found attitudes toward the protection of the Andean bear as a predictor of the desires for liking in social media (β = 0.48, *p* < 0.001), but neither for the desires for donating money (β = 0.03, *p* > 0.05) nor volunteering (β = 0.11, *p* > 0.05), despite the correlations between these variables. In a similar manner, subjective norms were a predictor of desires for liking (β = 0.16, *p* < 0.05), but not for money donation (β = 0.07, *p* > 0.05) and volunteering (β = 0.12, *p* > 0.05). Interestingly, perceived behavioral control was a predictor for the desires of liking (β = 0.14, *p* < 0.05) as well as the desires for money donation (β = 0.68, *p* < 0.001) and volunteering (β = 0.69, *p* < 0.001).

Similarly, perceived behavioral control also remained the strongest predictor in the second step of the regressions (model 2 in Table 3) because it was the strongest predictor for the desires for money donation (β = 0.59, *p* < 0.001) as well as volunteering (β = 0.61, *p* < 0.001). For these two behaviors, past donations (β = 0.20, *p* < 0.001) and past volunteering (β = 0.12, *p* < 0.05) emerged as further predictors stemming from the MGB. Enjoyment was the only independent variable that explained variance for the desires for liking (β = 0.22, *p* < 0.01), money donation (β = 0.24, *p* < 0.001), as well as volunteering (β = 0.30, *p* < 0.001). Similar to the first step, attitudes only predicted the desires for liking (β = 0.35, *p* < 0.05), but not for money donation (β = −0.03, *p* > 0.05) and volunteering (β = 0.03, *p* > 0.05).

In the third regression step, we investigated the possibility of using the dependent variables as predictors of each other. We found predictive abilities of the desires for liking for both of the classical environmental behaviors of money donation (β = 0.11, *p* < 0.01) as well as volunteering (β = 0.19, *p* < 0.01). Whereas money donation was not predictive for liking (β = 0.07, *p* > 0.05), volunteering was the only predictor of liking (β = 0.25, *p* < 0.001). Given the similarity between the classical behaviors, volunteering was a strong predictor of money donation (β = 0.68, *p* < 0.001) and vice versa (β = 0.61, *p* < 0.001). These differences were also reflected by the explained variances due to the fact that the classical behaviors were explained with 54% (R^2^_DON_ = 0.54) and 51% (R^2^_VOL_ = 0.51), in contrast to the desires for environmental liking, for which with 14% only a small amount of variance was explained (R^2^_LIKE_ = 0.14). In particular, these differences in the explained variances illustrate how liking may rather be an independent variable for the classical behaviors as the other way around. Based on these connections between the variables, liking was therefore integrated as a predictor of the other environmental behaviors. Environmental liking retained its predictive abilities in the final step, which included all previous variables as well as the demographic variables of age and gender. In this full model (model 4 in Table 3), attitudes (β = 0.34, *p* < 0.01), gender (β = −0.32, *p* < 0.001), enjoyment (β = 0.21, *p* < 0.01), as well as perceived behavioral control (β = 0.14, *p* < 0.05) predicted the desires for liking. In contrast, money donation was only predicted by perceived behavioral control (β = 0.55, *p* < 0.001), past donations (β = 0.20, *p* < 0.001), enjoyment (β = 0.19, *p* < 0.001), as well as liking (β = 0.14, *p* < 0.01). These independent variables (except of past behavior) also predicted the desires for volunteering, which was also predicted by perceived behavioral control (β = 0.58, *p* < 0.001), enjoyment (β = 0.25, *p* < 0.001), and the desires for liking (β = 0.17, *p* < 0.001). These results additionally underline the theoretical similarities between money donation and volunteering, but especially the differences of these more classical behaviors to the desires for liking.

Concerning the proportion of explained variance, the full model explained the highest rate of variance within all dependent variables (*R*^2^ = 0.35–0.48). Further, the differences between the explained variance of models 1 and 2 were rather small because the second step only explained between 2 and 4% more of the variance within the dependent variables. Nonetheless, we found differences in the predictive abilities of the corresponding independent variables, illustrating theoretical differences between the behaviors. Concerning these differences, all models consistently explained the least proportion of variance within the desires for liking, a medium amount within the desires for donating money, and the highest amount for the desires for volunteering.

### Correlations and Regressions Between Liking, Money Donation, and Volunteering (H_9–11_)

As described in Table 2 the correlations between the dependent variables resembled the suggested differences of environmental liking and the classical environmental behaviors. For example, desires for donating money and volunteering correlated more strongly with each other (*r* = 0.56, *p* < 0.01) than with the desires for environmental liking. Nonetheless, environmental liking was positively correlated with a small effect size to donating money (*r* = 0.26, *p* < 0.01) and with a medium effect size to volunteering (*r* = 0.35, *p* < 0.01).

Based on the predictive abilities of the desires for environmental liking for the classical behaviors, we further investigated the connections between the variables in two path models. Concerning the independent variables, we selected perceived behavioral control and enjoyment as predictors for all dependent variables, attitudes and gender as predictors for the desires for liking, and past behavior as well as the desires for liking as predictors of the desires for the classical behaviors of money donation and volunteering.

**FIGURE 2:**
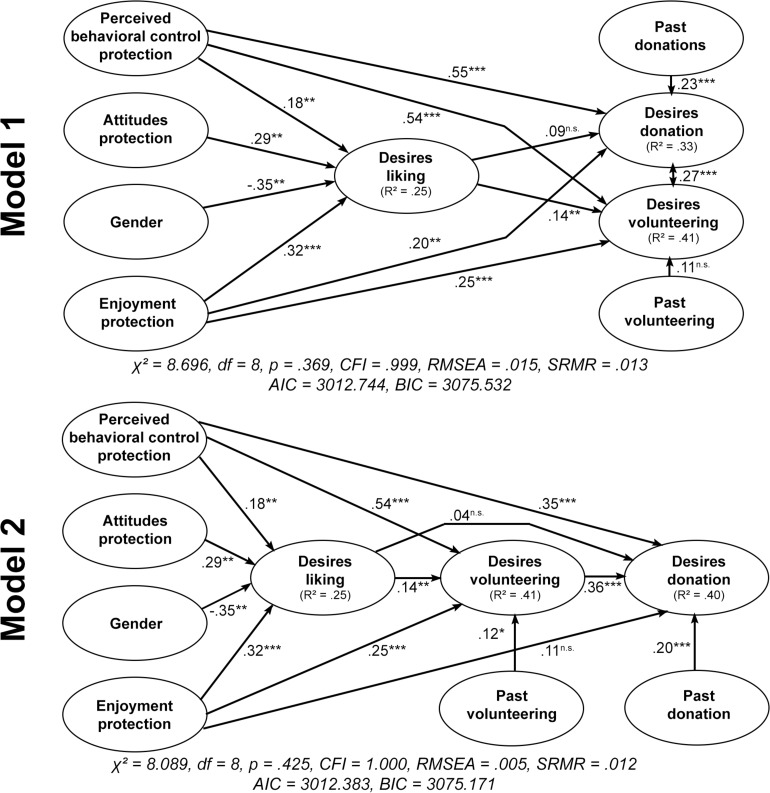
Comparison of path models with standardized regression coefficients of selected predictors from the model of goal-directed behavior and the desires for environmental liking as a direct predictor of the desires for money donation and volunteering **(model 1)** or the desires for environmental liking as a predictor of volunteering, which then predict money donation **(model 2)**. Gender was coded with female (1) and male (2). Model fit criteria: Chi-square (χ^2^), Degrees of freedom (*df*), Significance of chi-square value (*p*), Root mean square error of approximation (RMSEA), Bentler comparative fit index (CFI), standardized root mean square residual (SRMR), Akaike information criterion (AIC), Bayesian Information Criterion (BIC). ^n.s.^*p* > 0.05, ^∗∗^*p* < 0.01, ^∗∗∗^*p* < 0.001. *R*^2^ = explained variance within the corresponding dependent variable.

As displayed in model 1 of Figure 2, the model showed sufficient fit to the data and we found gender (β = −0.35, *p* < 0.01), enjoyment (β = 0.32, *p* < 0.001), attitudes (β = 0.29, *p* < 0.01), as well as perceived behavioral control (β = 0.18, *p* < 0.01) as predictors of the desires for liking. Whereas these variables explained 25% of the variance within the desires for liking (R^2^_LIKE_ = 0.25), perceived behavioral control was also a strong positive predictor of the desires for money donation (β = 0.55, *p* < 0.001) as well as volunteering (β = 0.54, *p* < 0.001). Similarly, enjoyment also predicted the desires for money donation (β = 0.20, *p* < 0.01) as well as volunteering (β = 0.25, *p* < 0.001). But in contrast to the prior regressions, desires for liking only predicted the desires for volunteering (β = 0.14, *p* < 0.01), but not for money donation (β = 0.09, *p* > 0.05). Additionally, past donations predicted the desires for money donation (β = 0.23, *p* < 0.001), and the desires for money donation as well as volunteering were correlated with each other (*r* = 0.27, *p* < 0.001). These differences in the predictive abilities of the independent variables resulted in differences in the explained variance because the variance within the desires for money donation (R^2^_DON_ = 0.33) showed a smaller amount of explained variance than the desires for volunteering (R^2^_VOL_ = 0.41).

Based on these effects of the desires for environmental liking, we calculated a final path model (model 2 in Figure 2). In this path model, we hierarchically ordered the dependent variables from environmental liking in social media to volunteering and money donation as the final dependent variable. This model showed a better fit to the data than model 1 (higher CFI, lower RMSEA) and explained more variance within the desires for environmental money donations (R^2^_DON_ = 0.40). Nearly all effects remained the same, except for the desires for volunteering, which now predicted the desires for money donation (β = 0.36, *p* < 0.001). The desires for environmental liking on social media still predicted the desires for volunteering (β = 0.14, *p* < 0.01) and were even a stronger predictor than past volunteering (β = 0.12, *p* < 0.05), but similar to the first model, liking still did not predict the desires for money donation (β = 0.04, *p* > 0.05). This second model shows how liking may be predictive for specific forms of environmental activism with a moderate difficulty, which then may lead to further behaviors due to possible spillover effects.

## Discussion

### Sharpening the Theory of Environmental Social Media Behavior (RQ_1_)

A further theory of environmental social media behavior is an open research question (Büscher, 2016) but may be important for the further mainstreaming of sustainability (Pearson et al., 2016). Within our first research question, which investigated the antecedents of the corresponding desires for the environmental behaviors, we utilized the socio-psychological MGB and compared the predictive effects of the independent variables, to get a better insight on the theoretical differences and similarities between the desires for the environmental behaviors. We organize the discussion of these differences and similarities based on the order of hypotheses, starting with the classical variables from the TPB (H_1–3_), then proceeding with the additional variables from the MGB (H_4–6_), and finally discussing the investigated demographic characteristics of gender (H_7_) and age (H_8_).

Starting with our first hypothesis, the effect of attitudes on the desires, we generally only found predictive effects of attitudes for the desires for liking on social media, but not the desires for the classical environmental behaviors (H_1_). When enjoyment was entered as a predictor in the second step, attitudes lost some of their predictive abilities. This indicates how enjoyment replaced attitudes for the explanation of the affective dimension of protection. This replacement may be inferred from the only small change in explained variance when the MGB variables were added in combination with a decrease of the regression coefficient of attitudes. Attitudes always include affective, cognitive, and a behavioral perspective (Ajzen, 1991), indicating how enjoyment may be a stronger and more behaviorally relevant affective predictor of desires than attitudes. This work adds to the discussion of affective variables within the framework of the TPB (Ajzen, 2011), but should only carefully be abstracted to other contexts because there may be contexts with similar predictive abilities of attitudes as well as emotions (Carrus et al., 2008). Generally, this result is in line with studies about emotions within social media (Brady et al., 2017), which is why this area should be further elaborated in future studies. One example might be the explicit confrontation of people with emotional stimuli from social media based on pictures or comments referring to environmental topics. These studies may further illustrate how social media could lead to attitudinal change and therefore be a way of fostering further environmental activism.

As another variable from the TPB, subjective norms also demonstrated to possess only minor predictive abilities, despite their bivariate correlations to all desires (H_2_). This observation partly contradicts prior experimental studies, which showed how the formation of group identities may be important for environmental behavior in digital media (Buhrmester et al., 2018). This result may be attributed to the rather open measurement of subjective norms, which is in line with prior studies within the framework of the MGB (Song et al., 2012), but may not focus specifically enough on the phenomenon of commenting posts in social networks relating to the conservation of the Andean bear or other social cues in social media. Social cues have been found to strongly affect social media behavior, at least within the context of political mobilization (Bond et al., 2012). For future studies about environmental social media, the clues about subjective norms could be strengthened, for example people could be explicitly confronted with comments of other people within the context of study, similar to the study of Bond et al. (2012).

Concerning perceived behavioral control, the final variable of the TPB, we found major differences between the desires for the corresponding environmental behaviors (H_3_). Perceived behavioral control was a significant predictor of all three desires, but the regression coefficients for the classical environmental behaviors of money donation and volunteering were severely larger than for the desires for liking on social media. While this observation may illustrate how liking as an example social media behavior may require fewer personal resources than other environmental behaviors, this result partly contradicts prior studies, which found no predictive effect of perceived behavioral control for example on money donation intentions (Kashif et al., 2015). But as this study used another, non-student sample, we believe that the missing personal resources of our participants might explain why perceived behavioral control showed such strong effects for the motivation to donate money and to volunteer. Based on the absence of such a strong predictive effect on the desires for liking on social media, social media could be an interesting entry point for fostering environmental action, particularly within populations with limited personal resources. As we will discuss later in concordance with other studies (Baker and White, 2010), this easiness may indeed be useful for conservation practice because liking may be inevitable for further distributing specific conservation-related contents in social media (van Dijck and Poell, 2013). For this further distribution of contents other factors from the subsequent MGB showed to be more important than the presented variables from the TPB.

Speaking of our first hypothesis from the MGB, we found enjoyment toward the protection of the species as the only consistent predictor for all desires in the full model (H_4_). While this result underlines the importance of emotions for environmental behavior in line with prior research (Caissie and Halpenny, 2003; Kals and Müller, 2012), the predictive ability may be also attributed to the specific context of our study, with prior studies showing how emotions play an important role in species conservation (Jacobs et al., 2014). Nonetheless, especially based on the results from the step-wise regression, emotions may be the most important affective determinant of self-reported environmental action. At least based on our results, positive emotions such as enjoyment showed to be more relevant for the desires to fulfill environmental behaviors than negative emotions.

This result was explicitly shown in the regressions as well as path models, in which the negative emotion of anger showed no predictive abilities, even when it was correlated with the desires (H_5_). Interestingly, anger was more strongly correlated with liking than with the other environmental desires. This may be explained as the anger caused by an unwillingness to protect the species can be more easily channeled by liking than with donating money or volunteering. Nonetheless, as with the effects of subjective norms the results for anger should only cautiously be generalized, as our study was in a rather general nature. For example, it would be possible in future studies to more explicitly investigate anger as a predictor of liking negative material in social networks. In this regard, a significant effect of anger may be likely and important for better understanding negativity in social networks. But for the present study, we rather took the view of protecting nature to explain how motivation for protection the Andean bear may be fostered.

Concerning the final MGB variable, the desires also differed based on the predictive effect of past behavior, which only affected the motivations to donate money and to volunteer for the protection of the Andean bear, but not liking on social media (H_6_). This result partly contrasts prior research about social media behavior which was not predicted by past behavior (Pelling and White, 2009), but is in line with prior research regarding money donation which found past behavior as the strongest predictor of future donation (Kashif et al., 2015). This relation was also shown by our data because the correlations between past behavior and the desires were stronger for the environmental behavior of donating money than for the other behaviors. We believe that the missing effect for the desires for liking might be explainable by the prevalent connection of the other MGB variables, particularly of enjoyment and gender, which were not predictors in the study of Pelling and White (2009), but explained a large amount of variance in the present work.

While this result underlines the motivational relevance of these factors, future studies could investigate the connection and causality between past and social media behavior based on an experimental comparison between people who have or have not supported an environmental organization on social media concerning their protection motivation. For such research, the integration of real social media data would also be interesting because the reliance on self-report measures in our study may have biased the results due to social desirability. While the high means indicate such a response pattern within our sample, we mainly interpreted the correlations and predictive abilities between the behaviors, which showed to be more stable across sample than mean differences. Therefore, even when some variables were skewed, our results are nonetheless of interest for environmental social media theory. Besides the already presented results concerning the hypotheses from the TPB and MGB, we also found interesting results regarding the demographic variables of gender and age. The predictive ability of gender only for the desires for liking on social media in particular adds knowledge to the theory of environmental social media behavior (H_7_). Prior studies have found a higher environmental motivation for female participants (Gifford and Nilsson, 2014; Olsson and Gericke, 2017), but only within the context of social media was this effect replicated in our study. Whereas the effect of gender was already reported in an evaluation study within the context of the Andean bear (Espinosa and Jacobson, 2012), gender only affected the desires for environmental liking, but not the desires for money donation or volunteering. This result is consistent with other studies, which found gender as a differentiating factor for general social media behavior (Schwartz et al., 2013). Because we only tested the behavior of liking in the case of protecting the Andean bear, this effect of gender on environmental social media behavior should only cautiously be generalized. But based on the prior results, gender seems nonetheless to be of interest for environmental social media behavior.

Concerning the second demographic control variable, we found no effect of age on any of the desires (H_8_). This result may be attributed to the small variance within the sample, and future studies could investigate how social media behavior may be especially interesting for the younger generation, which shows a close relationship to new technologies (Aksoy et al., 2013). This would be interesting for further studies.

### Liking as a Pathway to Species Conservation Action (RQ_2_)

Concerning correlational and predictive results between the desires for the environmental behaviors (RQ_2_), we found correlations between the desires to like for the protection of the Andean bear and the desires to donate money and to volunteer (H_9_), which were stronger between the classical behaviors (H_10_). The desires for liking also showed predictive abilities for the desires for money donation and volunteering in the regression analysis. This result is in line with our hypothesis and in line with prior research (Pearson et al., 2016). But while the regressions seemed promising concerning the effects of liking on the more difficult environmental behaviors, the calculated path models allowed for a more pronounced view.

Within this perspective, liking indeed may be connected to the desires for more difficult environmental behaviors, but be nonetheless only of limited reach. This hypothesis was in fact supported because liking predicted the desires for volunteering, but not for money donation (H_11_). Because money donation was generally the least desired behavior, this result may further clarify the role of liking as a possible spillover behavior (Nilsson et al., 2017).

For the concrete application within conservation, this observation would imply online campaigns as a possible low-level entry point for environmental action, which nonetheless remains only of limited reach for transformative societal actions (Halupka, 2014). Hence, every form of social media behavior should sooner or later be translated into further, real-world actions. Because our data indicated liking as a possible proxy of such a transformation into real-world behavior, attention of people in online campaigns can be the foundation for further conservation work and should therefore have a place in the higher rationale of species conservation (Parsons, 2016). Future studies may concentrate on this connection and clarify factors which facilitate the spill-over from liking to more difficult environmental behaviors (Nilsson et al., 2017).

Overall, successful conservation largely depends on successful communication, in a similar manner to classical activism. But especially for the issue at hand, social media could be an important way of wider information distribution, with prior studies revealing a lack of information about the Andean bear as a serious problem for the conservation of the species (Can et al., 2014). For future environmental work, it may be interesting to further investigate possible spillover effects of social media to other behaviors (Nilsson et al., 2017). In particular, studies regarding the impact of social media behavior may provide a way to overcome the often-discussed intention-behavior gap (Sheeran, 2002).

In this regard, our results also underline the role of perceived behavioral control as a relevant underlying predictor of more demanding environmental behaviors. This work is in line with prior research and should be kept in mind when designing intervention studies, which may be not successful if they only address one specific factor, such as identity (Fanghella et al., 2019).

## Conclusion

As described above, our study enabled for a comparison between the desires of liking on social media and two other more classical environmental behaviors, and found specific differences and connections based on the selected theoretical framework. Whereas the participants were highly motivated for the protection of the Andean bear in social media, smaller motivations were reported for volunteering and money donation. We were able to explain these differences based on the absence of a predictive effect of perceived behavioral control on the liking motivation, which we interpreted as the evaluation of this behavior to be easier. We also found gender to be a second major difference because this variable only affected social media protection behavior. Only enjoyment coherently predicted variance within all environmental behaviors.

Besides these variable specific connections between the independent variables from the MGB, the results demonstrated how this general socio-psychological model better explained the classical environmental behaviors than the innovative environmental behavior of liking on social media. This observation seems rather arbitrary, but indicates how traditional socio-psychological models may be only of limited applicability to these new contexts of psychological behavior. This result may be explainable by the nature of social media, which may be determined by further more egoistic reasons because several studies have illustrated how the construction of self-status or entertainment may affect social media behavior (Khan, 2017). Therefore, our research further developed but also questioned recent approaches of theorizing (environmental) social media behavior. For example, we found connections between liking and other more costly environmental behaviors. Future studies now could further elaborate on these connections and differentiate between specific platforms, as other studies have also done (Waterloo et al., 2018).

Concerning the limitations of our work, we critically discussed the reliance on self-report as well as the correlational design of the study. Future analyses should try to integrate real-world data to further deepen the understanding of social media behavior and possible connections with donations, but such studies require a basic understanding regarding the behaviors being analyzed. Our work aimed for such a foundational insight into the antecedents for and correlations between the behaviors under study.

Although all the mentioned variables should be reflected in future studies as well as the development of a theory of environmental social media behavior (Büscher, 2016), the connection of the desires for liking with the other more demanding desires for environmental behaviors seems particularly promising for expanding sustainable development. Nonetheless, future work needs to investigate the role of digital places for sustainable development (Gifford, 2014) and how the framing within these digital places might lead to real-world change (Lakoff, 2010). As our study demonstrated, environmental social media activity may lay the foundation for more difficult environmental behavior. This foundation may then entail further environmental action, for example by enabling group and identity building, as shown in a case study about donator identity formation against lion hunting (Buhrmester et al., 2018).

In concordance with these studies, our results illustrated the evidence for hypothesizing connections between environmental liking and real-life environmental behavior. Therefore, these further studies may contribute to a better understanding of modern day environmental behavior and uncover how social media can facilitate pro-environmental behavior, which may one day lead to the required more sustainable societies.

## Data Availability

All datasets generated for this study are included in the manuscript and/or the Supplementary Files.

## Ethics Statement

This study was carried out in accordance with the relevant national guidelines and laws of the study country, the selected university, the Declaration of Helsinki as well as APA’s Code of Conduct to ensure compliance to all ethical and legal standards. This included, for example, the guarantee of anonymity and participation on voluntary basis, as all participants had the chance to decline their participation at any time without negative consequences. Furthermore, we assured informed consent by giving written information on the first page and verbal information prior to the sampling. We therefore ensured consensus about the purpose and aims of the study between all participants and implied this informed consent by survey completion (Nijhawan et al., 2013). Due to the non-medical background, the absence of personal risks and the full awareness about the purpose and aims of all participants, an ethics approval was not required by institutional, national, and international guidelines (Nijhawan et al., 2013).

## Author Contributions

AB conceptualized and designed the study, performed the statistical analysis, and wrote the first draft of the manuscript. AT performed the investigation and gathered the data. AB and SM reviewed and edited the manuscript.

## Conflict of Interest Statement

The authors declare that the research was conducted in the absence of any commercial or financial relationships that could be construed as a potential conflict of interest.

## Footnotes

1 https://www.facebook.com/AndeanBearFoundation/

2 https://twitter.com/BearAndean
