# Supplementary material for: Can a Like Save the Planet? Comparing Antecedents of and Correlations Between Environmental Liking on Social Media, Money Donation, and Volunteering
Source: Front Psychol. 2019 Aug 28;10:1989. doi: 10.3389/fpsyg.2019.01989 (PMC6722222; doi:10.3389/fpsyg.2019.01989)
Supplement: Supplementary file 1 [file Data_Sheet_1.ZIP › Variable description.pdf]

Supplementary Material

1 Variable description

#	Variable name in data set	Description/Wording (English - Spanish)	Coding	Comment
**Demographic data**				
1	ID	Identification number of Participants		The number was assigned after the data collection and is not connected to specific persons
2	Age	Age of participants	Open<br>99 = missing value	
3	Gender	Gender of participants	1 = female<br>2= male<br>99 = missing value	
	Degree	Intended degree of participants	Open<br>99 = missing value	Not included in data set due to the protection of anonymity
**Attitudes Protection**				
4	ATT01	I think the conversation of the Andean bear is a positive attitude. – Pienso que la conservación del oso andino es una actitud positiva.	1 = do not agree at all<br>2 = do not agree<br>3 = even agree<br>4 = rather agree<br>5 = agree<br>6 = agree completely<br>99 = missing value	
5	ATT02	I think the conservation of the Andean bear is a useful attitude. - Pienso que la conservación del oso andino es una actitud útil.	1 = do not agree at all<br>2 = do not agree<br>3 = even agree<br>4 = rather agree<br>5 = agree<br>6 = agree completely<br>99 = missing value	
6	ATT03	I think the conservation of the Andean bear is a valuable attitude. - Pienso que la conservación del oso andino es una actitud valiosa.	1 = do not agree at all<br>2 = do not agree<br>3 = even agree<br>4 = rather agree<br>5 = agree<br>6 = agree completely	

#	Variable name in data set	Description/Wording (English - Spanish)	Coding	Comment
			99 = missing value	
**Enjoyment Protection**				
If I promoted the conservation of the Andean bear, I would feel… - Si apoyara a la conservación del oso andino, me sentiría…				
7	ENJ01	… happy. – feliz.	1 = do not agree at all<br>2 = do not agree<br>3 = even agree<br>4 = rather agree<br>5 = agree<br>6 = agree completely<br>99 = missing value	
8	ENJ02	… satisfied. – satisfecho/a.	1 = do not agree at all<br>2 = do not agree<br>3 = even agree<br>4 = rather agree<br>5 = agree<br>6 = agree completely<br>99 = missing value	
9	ENJ03	… delighted. – deleitado/a.	1 = do not agree at all<br>2 = do not agree<br>3 = even agree<br>4 = rather agree<br>5 = agree<br>6 = agree completely<br>99 = missing value	
**Anger Protection**				
If I promoted the conservation of the Andean bear, I would feel… - Si encontrara un oso andino en el páramo, me sentiria…				
10	ANG01	… angry. – enfadado/a.	1 = do not agree at all<br>2 = do not agree<br>3 = even agree<br>4 = rather agree<br>5 = agree<br>6 = agree completely<br>99 = missing value	
11	ANG02	… irritated. – irritado/a.	1 = do not agree at	

#	Variable name in data set	Description/Wording (English - Spanish)	Coding	Comment
			all<br>2 = do not agree<br>3 = even agree<br>4 = rather agree<br>5 = agree<br>6 = agree completely<br>99 = missing value	
12	ANG03	… furious. – enfadado/a.	1 = do not agree at all<br>2 = do not agree<br>3 = even agree<br>4 = rather agree<br>5 = agree<br>6 = agree completely<br>99 = missing value	
**Subjective Norms Protection**				
13	SN01	If I supported the conservation of the Andean bear, most of the people important to me would agree. – Si apoyara a la conservación del oso andino, la mayoría de las personas importantes para mí estaría de acuerdo.	1 = do not agree at all<br>2 = do not agree<br>3 = even agree<br>4 = rather agree<br>5 = agree<br>6 = agree completely<br>99 = missing value	
14	SN02	If I supported the conservation of the Andean bear, most of the people important to me would support me.– Si apoyara a la conservación del oso andino, la mayoría de las personas importantes para mí me apoyaría.	1 = do not agree at all<br>2 = do not agree<br>3 = even agree<br>4 = rather agree<br>5 = agree<br>6 = agree completely<br>99 = missing value	
15	SN03	If I supported the conservation of the Andean bear, most of the people important to me would understand me.– Si apoyara a la	1 = do not agree at all<br>2 = do not agree<br>3 = even agree<br>4 = rather agree<br>5 = agree<br>6 = agree completely	

#	Variable name in data set	Description/Wording (English - Spanish)	Coding	Comment
		conservación del oso andino, la mayoría de las personas importantes para mí lo entendería.	99 = missing value	
**Perceived Behavioral Control Protection**				
16	PBC01	I am able to support the conversation of the Andean bear. – Soy capaz de apoyar a la conservación del oso andino.	1 = do not agree at all<br>2 = do not agree<br>3 = even agree<br>4 = rather agree<br>5 = agree<br>6 = agree completely<br>99 = missing value	
17	PBC02	I have enough financial resources to support the conversation of the Andean bear. – Tengo los suficientes recursos económicos para apoyar a la conservación del oso andino.	1 = do not agree at all<br>2 = do not agree<br>3 = even agree<br>4 = rather agree<br>5 = agree<br>6 = agree completely<br>99 = missing value	
18	PBC03	I have enough time to support the conversation of the Andean bear. – Tengo el suficiente tiempo para apoyar a la conservación del oso andino.	1 = do not agree at all<br>2 = do not agree<br>3 = even agree<br>4 = rather agree<br>5 = agree<br>6 = agree completely<br>99 = missing value	
**Past Behaviors**				
How often did you… - ¿Cuantas veces usted ya ha…				
19	PBNET	… give a "Like" through social networks to organizations that conserve the Andean bear? - … dado me gusta por medio de redes sociales a organizaciones que conservan al oso andino?	1 = never<br>2 = rarely<br>3 = occasionally<br>4 = often<br>5 = very often<br>99 = missing value	Past behavior for promotions in a social network; Was directly used for the regressions, as it was one item
20	PBDON	… donate money for	1 = never	Past behavior for money

#	Variable name in data set	Description/Wording (English - Spanish)	Coding	Comment
		the conversation of the Andean bear? - … donado para la conservación del oso andino?	2 = rarely<br>3 = occasionally<br>4 = often<br>5 = very often<br>99 = missing value	donation; Was directly used for the regressions, as it was one item
21	PBVOL	… volunteer to support foundations such as the "Fundación Oso Andino" - … hecho un voluntariado para apoyar a fundaciones como la "Fundación Oso Andino"?	1 = never<br>2 = rarely<br>3 = occasionally<br>4 = often<br>5 = very often<br>99 = missing value	Past behavior for volunteering; Was directly used for the regressions, as it was one item
**Desires Social Network**				
22	DESNET01	I would gladly "like" organizations that conserve the Andean bear in a social network. – Me gustaría darle me gusta por medio de redes sociales a organizaciones que conservan al oso andino.	1 = do not agree at all<br>2 = do not agree<br>3 = even agree<br>4 = rather agree<br>5 = agree<br>6 = agree completely<br>99 = missing value	
23	DESNET02	I wish to "like" organizations that conserve the Andean bear in a social network. – Deseo darle me gusta por medio de redes sociales a organizaciones que conservan al oso andino.	1 = do not agree at all<br>2 = do not agree<br>3 = even agree<br>4 = rather agree<br>5 = agree<br>6 = agree completely<br>99 = missing value	
24	DESNET03	I want to "like" organizations that conserve the Andean bear in a social network. – Quiero darle me gusta por medio de redes sociales a organizaciones que conservan al oso andino.	1 = do not agree at all<br>2 = do not agree<br>3 = even agree<br>4 = rather agree<br>5 = agree<br>6 = agree completely<br>99 = missing value	
**Desire Donation**				
25	DESDON01	I would gladly donate money for the conservation of the	1 = do not agree at all<br>2 = do not agree	

#	Variable name in data set	Description/Wording (English - Spanish)	Coding	Comment
		Andean bear. – Me gustaría donar para la conservación del oso andino.	3 = even agree<br>4 = rather agree<br>5 = agree<br>6 = agree completely<br>99 = missing value	
26	DESDON02	I wish to donate money for the conservation of the Andean bear. – Deseo donar para la conservación del oso andino.	1 = do not agree at all<br>2 = do not agree<br>3 = even agree<br>4 = rather agree<br>5 = agree<br>6 = agree completely<br>99 = missing value	
27	DESDON03	I want to donate money for the protection of the Andean bear. – Quiero donar para la conservación del oso andino.	1 = do not agree at all<br>2 = do not agree<br>3 = even agree<br>4 = rather agree<br>5 = agree<br>6 = agree completely<br>99 = missing value	
	**Desire Volunteering**			
28	DESVOL01	I would gladly support foundations like “Fundación Oso Andino” by volunteering. – Me gustaría hacer un voluntariado para ayudar a organizaciones como la “Fundación Oso Andino”.	1 = do not agree at all<br>2 = do not agree<br>3 = even agree<br>4 = rather agree<br>5 = agree<br>6 = agree completely<br>99 = missing value	
29	DESVOL02	I wish to support foundations like “Fundación Oso Andino” by volunteering. – Deseo hacer un voluntariado para ayudar a organizaciones como la “Fundación Oso Andino”.	1 = do not agree at all<br>2 = do not agree<br>3 = even agree<br>4 = rather agree<br>5 = agree<br>6 = agree completely<br>99 = missing value	
30	DESVOL03	I would like to support	1 = do not agree at	

#	Variable name in data set	Description/Wording (English - Spanish)	Coding	Comment
		foundations like “Fundación Oso Andino” by volunteering. – Quiero hacer un voluntariado para apoyar a organizaciones que conservan al oso andino.	all<br>2 = do not agree<br>3 = even agree<br>4 = rather agree<br>5 = agree<br>6 = agree completely<br>99 = missing value	
**Aggregated variables**				
31	ATTPROT	Attitudes towards the protection of the Andean bear	Mean value from items ATT01-03<br>99 = missing value	
32	ENJPROT	Enjoyment towards the protection of the Andean bear	Mean value from items ENJ01-03<br>99 = missing value	
33	ANGPROT	Anger towards the protection of the Andean bear	Mean value from items ANG01-03<br>99 = missing value	
34	SNPROT	Subjective norms towards the protection of the Andean bear	Mean value from items SN01-03<br>99 = missing value	
35	PBCPROT	Perceived behavioral control towards the protection of the Andean bear	Mean value from items PBC01-03<br>99 = missing value	
36	DESNET	Desires to promote the protection of the Andean bear in a social network	Mean value from items DESNET01-03<br>99 = missing value	
37	DESDON	Desires to donate money for the protection of the Andean bear	Mean value from items DESDON01-03<br>99 = missing value	
38	DESVOL	Desires to volunteer for the protection of the Andean bear	Mean value from items DESVOL01-03<br>99 = missing value	
